# Characteristics of physicians receiving large payments from pharmaceutical companies and the accuracy of their disclosures in publications: an observational study

**DOI:** 10.1186/1472-6939-13-24

**Published:** 2012-09-26

**Authors:** Susan L Norris, Haley K Holmer, Lauren A Ogden, Brittany U Burda, Rongwei Fu

**Affiliations:** 1 Department of Medical Informatics and Clinical Epidemiology, Oregon Health & Science University, 3181 SW Sam Jackson Park Road, Mail Code BICC, Portland, OR, 97239, USA; 2 Kaiser Permanente Center for Health Research, Portland, OR, USA; 3 Department of Public Health and Preventive Medicine, Oregon Health & Science University, Portland, OR, USA; 4 Department of Emergency Medicine, Oregon Health & Science University, Portland, OR, USA

**Keywords:** Conflict of interest, Publication, Industry relationships, Physicians, Bias, Disclosure, Accuracy

## Abstract

**Background:**

Financial relationships between physicians and industry are extensive and public reporting of industry payments to physicians is now occurring. Our objectives were to describe physician recipients of large total payments from these seven companies, and to examine discrepancies between these payments and conflict of interest (COI) disclosures in authors’ concurrent publications.

**Methods:**

The investigative journalism organization, ProPublica, compiled the Dollars for Docs database of payments to individuals from publically available data from seven US pharmaceutical companies during the period 2009 to 2010. We examined the cohort of 373 physicians in this database who each received USD $100,000 or more in the reporting period 2009 to 2010.

**Results:**

These physicians received a total of $52,600,624 during this period (mean payment per physician $141,020). The predominant specialties were internal medicine and psychiatry. 147 of these physicians authored a total of 134 publications in the first quarter of 2011 and 77% (103) of these publications provided a COI disclosure. 69% of the 103 publications did not contain disclosures of the payment listed in the Dollars for Docs database.

**Conclusions:**

With increased public reporting of industry payments to physicians, it is apparent that large sums are being paid for services such as consulting and peer education. In over two-thirds of publications where COI disclosures were provided, the disclosures by physician authors did not include industry payments that were documented in the Dollars for Docs database.

## Background

Financial relationships between physicians and industry are extensive in the U.S. [1,2], encompassing research funding to individuals and institutions, personal financial investments, and direct payments to physicians for services such as consulting, advising, speaking engagements, travel, and gifts. These financial relationships may represent conflicts of interest (COI) and thus can diminish the credibility of clinicians, researchers, research studies, and academic and other institutions that receive such payments. In addition, both funder and author conflicts are associated with bias in the results of research studies [3-7] and derivative products such as systematic reviews [8] and clinical practice guidelines [9].

Concern over COI and resultant risk of bias has engendered a variety of approaches and policies by biomedical journals, academic institutions, medical and continuing education institutions, healthcare delivery systems, professional organizations, and public sector policy makers. Central to COI policies is disclosure, and recent efforts to address COI have focused on disclosure and increased transparency. One such policy in the US is the Physician Payments Sunshine Act, signed into law in the US in 2010 as part of the Patient Protection and Affordable Care Act. This policy requires that pharmaceutical, medical device, biological, and medical supply manufacturers report to the U.S. Department of Health and Human Services payments that are more than $10 to physicians and teaching hospitals [10]. The law will require data reporting beginning in January 2013, including stock options, royalties, consulting fees, honoraria, education, research grants, meals, gifts, entertainment, and travel. In addition, the database will provide information on the physician receiving the payment, their address, payment date, and drug or device that the physician helped promote. There will be stiff penalties for both inadvertent lapses (up to $150,000 annually for failure to report) and intentional nondisclosure (up to $1 million annually) [10].

To date, drug companies have rarely disclosed payments to their speakers. Recently, however, a number of companies have begun posting doctors’ names and compensation on their web sites, some in anticipation of the Sunshine Act, others a result of legal settlements with the US government for illegal marketing of pharmaceuticals [11].

The objectives of this study were to describe the physician recipients of large total payments from pharmaceutical companies and to examine discrepancies between those payments and payee disclosures in concurrent publications. This study explores the feasibility, utility, and limitations of using publically available payment data to examine these discrepancies.

## Methods

The source of our payment data was the Dollars for Docs database, developed by ProPublica, an investigative journalism organization [11]. The Dollars for Docs database (referred to as “the database”) was first released October 18, 2010, containing about 30,000 discrete payments to approximately 17,000 US health care providers by seven pharmaceutical companies over varying numbers of quarters in 2009 and 2010. These seven companies represented 36% of prescription drug sales in the US in 2009 and disclosed $257.8 million in payments to US healthcare providers [11]. Five of these companies were required to make these disclosures by the US Justice Department (AstraZeneca, Cephalon, Eli Lilly, Johnson & Johnson, Pfizer); the other two (Merck, GlaxoSmithKline) released these data voluntarily in anticipation of the Sunshine Act.

ProPublica obtained these data from open-access websites for each of the seven companies and aggregated them into a single database. The original data were available in a variety of formats, including unsortable PDFs. ProPublica programming staff used a variety of programs to scrape and clean the data, including Google Refine [12], Firebug [13], Adobe Acrobat [14], and others [15] to import these data into spreadsheets. Names of payees varied, even within a single company’s dataset, and Google Refine was used to aggregate the data for each individual listed when variations in name occurred such as the addition of an initial or an abbreviated first name. We verified the aggregation of data by individuals in the Dollars for Docs database using the Massachusetts General Hospital Utility Multiprogramming System (MUMPS), reanalyzing the raw dataset provided by ProPublica, and confirmed that our cohort did receive the stipulated payments.

For each recipient of a payment from one of the seven companies, the database includes the recipient’s name, city and state, reason for the payment, company making the payment, and the quarter(s) when the payment was made. Companies varied in their categorization of the reasons for payments, for example AstraZeneca and Merck only reported payments to speakers, whereas Eli Lilly, Cephalon and Pfizer reported payments for a variety of services, including meals, travel, and consulting. The exact date for the payments was not provided, and companies varied in their aggregation of calendar quarters. The database has been continuously updated since its first release with the addition of more companies, individuals, and payments. For this study, we used payment data from the initial database, released in October, 2010.

We selected as our study cohort individuals who received more than USD $100,000 in the period 2009 to 2010. We chose this cohort of payees because the large amounts of funds that these individuals received should likely be remembered and disclosed in concurrent publications, and we felt that these payees may have characteristics that make them somewhat homogeneous in their behaviors. Among these individuals, we examined only physicians, excluding other healthcare providers. We obtained demographic information on the cohort, including age, sex, years since medical school graduation, primary affiliation, specialty, and state of medical board certification. These demographic data were obtained from institutional websites, HealthGrades© and a general Internet search. All data were entered into Microsoft Access (Redmond, WA).

In order to examine whether physicians disclose these payments, we identified publications by physicians in our cohort in any authorship position by searching Medline from January 1, 2011 through March 31, 2011 using the Ovid search engine. We included all types of publications (e.g., primary research studies, reviews, editorials), and confined our search to English language publications. Results were imported into an EndNote library (Carlsbad, CA). If we identified publications by authors with similar names, we reviewed the full text to further examine the institutional affiliation, credentials, and subject matter of the article to determine if the individual in our cohort authored the publication. If there was any uncertainty about whether a physician in our cohort authored a specific publication, a second member of our research team independently examined the publications and if there was disagreement, consensus was achieved.

We then compared the COI disclosures to payments made to those individuals as indicated in the Dollars for Docs database. Because the lag time between completion of the COI disclosure and the date of publication (both electronic and print) is variable and unknown, and the database does not contain exact payment dates, we compared disclosures in 2011 publications to payments received in 2009 and 2010. This should produce a conservative (low) estimate of discrepancies, as COI disclosures in publications in 2011 should in all likelihood include payments to those authors in the prior 2 years.

Since this is an observational, exploratory study, we did not perform sample size calculations. Descriptive statistics were used to summarize physician characteristics, total payments, and discrepancies in disclosures. An analysis of variance model was used to compare the mean total payments among specialties with adequate sample size (n > 20) and a linear regression model was used to assess the association between total payments and physician characteristics (age and sex). All analyses were conducted using SAS 9.3 (SAS Institute Inc., Cary, NC, USA).

## Results and discussion

**Figure 1:**
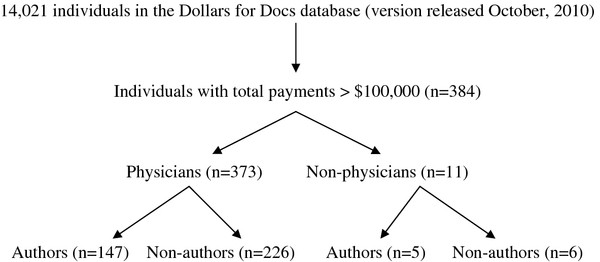
**Study flow diagram.** See text for details. Authors refer to individuals for whom we identified a publication within the period January to March 31, 2011.

Of the 18,297 individuals listed in the database, 384 were identified by ProPublica as earning $100,000 or more during the time period 2009 to 2010 (Figure 1). Of these 384 individuals, 373 (97%) were physicians; the remaining 11 were pharmacists, nurses, and other healthcare providers. The majority (89%) of these physicians were male and the average age was 53 years (median 53, range 36 to 75). The specialties most frequently represented in the cohort were internal medicine and psychiatry (each 31%).

The total payments to this physician cohort during the reporting period were $52,600,624, with a mean payment of $141,020 per physician (median $126,724, range $100,047 to $303,558) (Table 1). The mean total payment per physician per quarter for each company ranged from $21,305 for GlaxoSmithKline to $3596 for Johnson & Johnson. Payments were for consultation, continuing education presentation, and other services. None of the payments to this cohort of payees were categorized as research funding.

**Table 1:** Total payments and payments per physician by specialty

**Specialty**	**Number of physicians**	**Total payments (in USD)**	**Mean payment per physician (Standard Deviation) (In USD)**
**Primary care**	**145**	**21,286,443**	**146,803 (**44,767)
Family medicine	28	3,635,749	129,848 (35,017)
Pediatrics	2	244,800	122,400 (18,243)
Internal medicine	115	17,405,894	151,356 (52,078)
**Specialty medicine**	**75**	**10,955,643**	**146,075 (**38,979)
Allergy and Immunology	15	2,384,175	158,945 (34,934)
Cardiovascular disease	6	916,441	152,740 (35,098)
Endocrinology, Diabetes, and Metabolism	10	1,432,020	143,202 (36,229)
Hematology	3	374,300	124,767 (21,104)
Neurology	14	2,154,235	153,874 (52,078)
Obstetrics and gynecology	14	1,795,064	128,219 (26,031)
Oncology	1	240,150	240,150 (0)
Pain Medicine	2	225,429	112,715 (6,353)
Physical Medicine and Rehabilitation	6	852,103	142,017 (28,489)
Pulmonology	3	459,552	153,184 (58,778)
Rheumatology	1	122,174	122,174 (0)
**Surgery**	**24**	**3,689,339**	**153,722 (**38,167)
Otolaryngology	2	300,500	150,250 (35,285)
Surgery	4	585,380	146,345 (50,171)
Urology	18	2,803,459	155,748 (37,879)
**Psychiatry**	**117**	**14,710,749**	**125,733 (**23,936)
**Other**	**12**	**1,772,150**	**147,679 (**54,067)
Anesthesiology	8	1,397,329	174,666 (62,088)
Emergency Medicine	1	165,800	165,800 (0)
Nuclear Medicine	1	101,246	101,246 (0)
Radiology	1	186,300	186,300 (0)
Information not found	1	107,775	107,775
	**Total: 373**	**Total payments: 52,600,624**	**Mean payment per physician: 141,020 (39385)**

Payments are aggregated across the seven pharmaceutical companies.

*USD*, U.S. dollar.

For specialties with five or more physicians represented in our cohort, mean payments ranged from $125,733 for psychiatry to $174,666 for anesthesiology. Four specialties (family medicine, internal medicine, surgery, and psychiatry) had 20 or more physicians represented in our cohort and the mean total payments per physician per quarter were significantly different among these specialties (*P* < 0.0001). In particular, the mean total payment to physicians in internal medicine was significantly higher than to physicians in family medicine (mean difference $21,507; 95% confidence interval (CI) $1,533 to $41,482) and to psychiatrists (mean difference $25,623; 95% CI $13,176 to $38,069). The mean total payment to surgeons was also significantly higher than to psychiatrists (mean difference $27,990; 95% CI $6,750 to $49,229). The mean total payment to surgeons was not significantly different from that to physicians in internal medicine ($153,722 vs. $151,356). Based on results from the linear regression, the total payment per physician per quarter was not associated with sex (*P* = 0.1730) or age (*P* = 0.4908).

**Figure 2:**
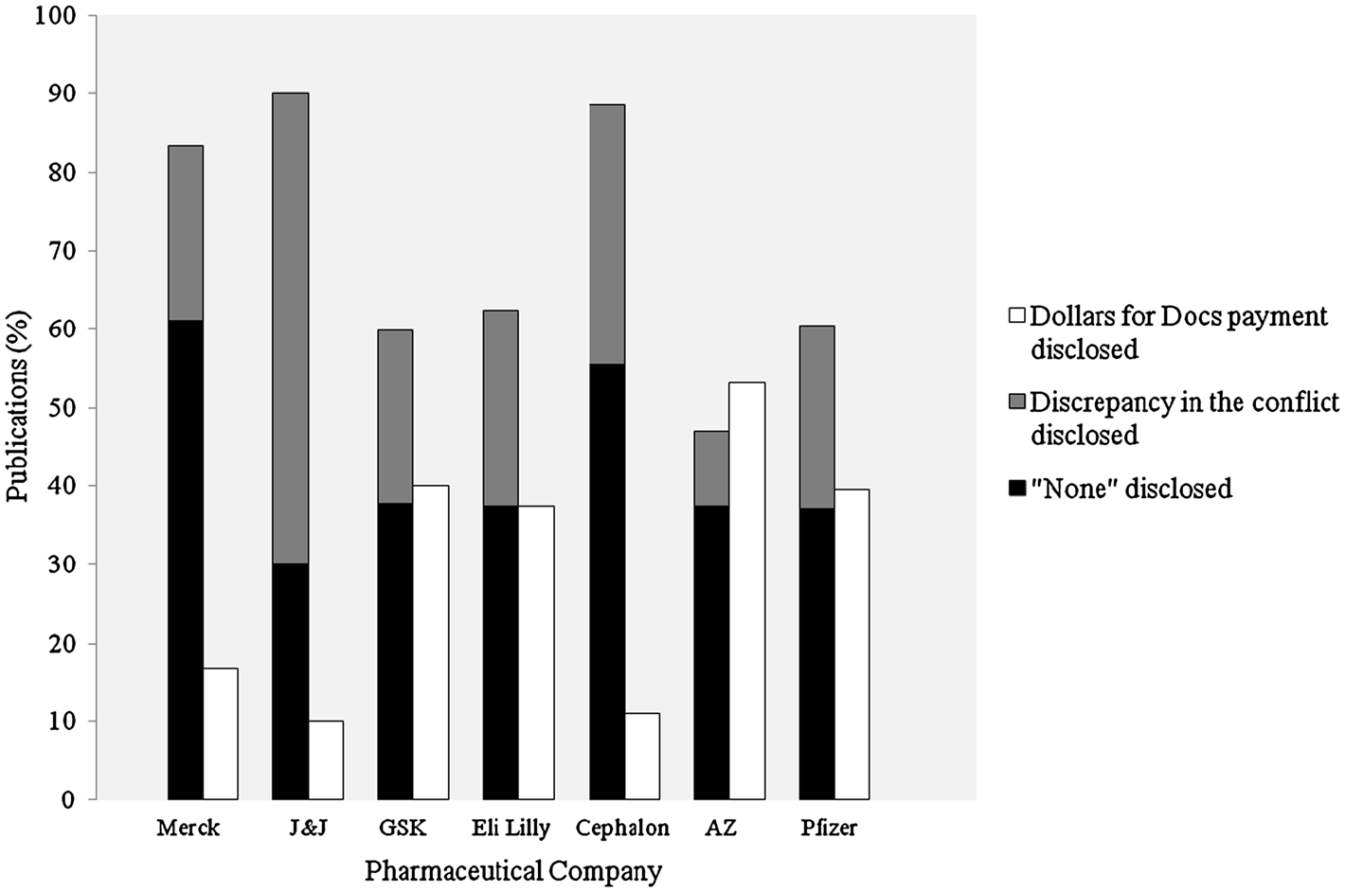
**The relationship between disclosures in publications and known payments by pharmaceutical companies to physician authors.** Abbreviations: AZ, AstraZeneca; GSK, GlaxoSmithKline; J&J, Johnson & Johnson. Both the grey and black bars represent discrepancies between the Dollars for Docs database and the physician author’s disclosures in publications. *Dollars for Docs payment disclosed* means that the specific company that made a payment to the physician was listed in the author’s disclosures in the publication. *Discrepancy in the conflict disclosed* means that one or more financial conflicts of interest were disclosed in the publication, but not the payer reported in the Dollars for Docs database. *No conflicts of interest disclosed* means that the publication stated that there were no conflicts of interest despite the Dollars for Docs database listing a payment.

147 of the 373 physicians published a total of 1223 articles between January 2009 and the end of March, 2011 (mean 8 publications per physician, median 4, range 1–125). Of the 134 publications by physicians in the first quarter of 2011, 103 (77%) provided author disclosures of COI in the publication while the remaining 23% did not. Among publications with disclosures, 42 (41%) reported that the index author had nothing to disclose, while in 29 publications (28%) the author disclosed a conflict other than the payment in the Dollars for Docs database. In the remaining 32 publications (31%), the payment in the database was disclosed. The percentage of publications with the payer in the Dollars for Docs database disclosed, stratified by the 7 companies, is displayed in Figure 2.

In summary, among a cohort of physicians who were known recipients of large total payments from a select group of 7 pharmaceutical companies, nearly one-quarter of their concurrent publications do not report any COI disclosures. Furthermore, of the publications with disclosure statements, over two-thirds do not report payments listed in the Dollars for Docs database.

Several prior studies have examined the accuracy of COI disclosures in journal publications, with similar results. Two recent studies compared disclosures in publications to payment information from an orthopedic device company. Chimonas and colleagues [16] reported high rates of nondisclosures: 50% among publications directly or indirectly related to payments. Okike and coauthors [17] reported rates of nondisclosure in published abstracts from an orthopedic meeting of 20.7% for payments that were directly related, and 50% for payments that were indirectly related to the topic of the abstract. Other studies comparing abstracts and subsequent publications or concurrent publications also suggest a high prevalence of inaccurate author disclosures [18,19].

Our study has important strengths. Our data originated with payer databases, which were placed in the public domain either voluntarily or by mandate of legal settlements. The use of physician payment data from pharmaceutical companies is novel, to our knowledge, and our study provides insights into the methods, feasibility, and limitations of using such datasets for studies of discrepancies between payments known to have occurred and disclosure in peer-reviewed journals. When datasets are more complete and journal policies more uniform, these approaches can be used to determine the accuracy and completeness of disclosures.

Our study findings have important implications. Journal editors and readers rely on accurate disclosures by authors in order to assess the credibility and risk of bias in journal publications, and our findings suggest that the accuracy and completeness of disclosures cannot be assumed at present. As industry payment data increasingly become publically available, however, journal staff and readers will be able to verify the accuracy of author disclosures. Though in the US, until the Sunshine Act is implemented and there is a unified public reporting system for industry payments to physicians, it may not be feasible for journal editors and staff to check author disclosures. For authors, the increasing availability of payment data may promote more accurate disclosures or may discourage physicians from accepting industry payments, although there is as yet no evidence to support these possibilities. It is hoped that the standardized reporting form of the International Committee of Medical Journal Editors [20] will improve the accuracy of disclosures, however such data are not yet available.

There are a number of limitations to this pilot work. First, these are exploratory analyses on a small cohort of American physicians, whose characteristics and behaviors are likely not representative of US physicians in general, including those who receive much smaller total payments or those who do not receive funds from industry. Second, the database that we examined was limited to the payments of only 7 pharmaceutical companies during variable intervals in 2009 and 2010. The exact dates for payments were not available, and the categorization of types of payments varied across companies, making comparisons of the reasons for payments difficult.

Third, we faced challenges identifying publications by our physician cohort: exact author names vary (e.g., use of middle initial or not) and can resemble names of non-cohort authors. If we failed to identify all of the author’s publications, we may bias our assessment of rates of discrepancies if missing publications have a different disclosure rate than the rate in the publications that we identified. There is a delay in indexing publications in Medline thus more recent publications may have been missed.

Fourth, our assessment of rates of discrepancies cannot be equated with inaccurate disclosures because of several assumptions that we made. We did not examine journal COI policies, but rather assumed that all journals required disclosures. We also did not examine how journal COI policies address relevance of the topic of the publication to the disclosure. We assumed that all payments in the database should have been disclosed in the identified publications.

It was difficult to determine the relationship between the time of disclosure and payments as it was not possible to know exactly when authors completed disclosures relative to the time of publication, as this varies widely across journals. Although some journals request an update of disclosures at the time of publication, it is unknown how frequently authors comply and how accurate those updates are. In addition, we only had information on the quarter or group of quarters when payments occurred, and not the exact dates. Although unlikely, we could have overestimated rates of discrepancies if disclosure occurred well prior to publication and prior to drug company payment, or if a disclosure was close to the publication date and the required time period of disclosure was short, with payment preceding the disclosure period. We feel that our approach of examining 2011 publications should minimize these biases, as disclosures in publications likely encompass payments in the prior 2 years. Chimonas and colleagues [16] used a similar approach when they compared payments to physicians by orthopedic device companies to disclosures in orthopedic publications 1 to 2 years after the payments were made.

Finally, we were unable to determine the reasons for discrepancies, which are undoubtedly multifactorial, including forgetting, carelessness, misreading or misinterpreting COI policies, lack of clarity in disclosure forms, or purposeful nonreporting. From these retrospective, observational data we clearly cannot determine causality or impute motive.

Future research can build on this work. Studies could further describe the characteristics of physicians and other healthcare professionals who accept payments across a broad range of values and payment types. The effectiveness of specific COI policies and disclosure forms for achieving accurate and complete disclosures should be explored for journals, institutional review boards, academic institutions, and other organizations. Predictors of the accuracy of COI disclosures can be examined when more complete payment data are available with implementation of the Sunshine Act [10]. The effect of public disclosure of industry payments may be linked to provider behavior such as the future acceptance of industry payments or the prescription of specific medications when individual prescribing data are available.

Little is known about the effect of disclosure policies and practices on the intended outcomes of improving transparency and aiding in the interpretation of journal articles. The dominant opinions in the literature are that disclosures are essential because they enable the “readers to form their own opinions on whether a conflict exists and what relevance it has for the study” [21]. However the evidence base for this opinion is scant. Readers may find an article less interesting and valid [22] and a trial less believable [23] if relevant industry relationships are disclosed. On the other hand, when presented with a direct comparison of an abstract with and without an industry conflict disclosed, physician readers did not differ significantly in their reported likelihood of prescribing the new drug [24]. Further work is thus needed to determine how disclosures are interpreted and the information used by the reader, and what constitutes optimal presentation, level of detail, and relevance.

## Conclusions

Transparency of industry payments to US physicians is increasing because of new and anticipated government policies and practices. Large amounts of money are going to physicians from industry for non-research-related services. In a small and select cohort of physicians receiving large total payments from pharmaceutical companies, there are discrepancies between payments known to have occurred and disclosures in publications. Journal editors need to continue to develop policies and approaches that lead to accurate and complete disclosures. Failure to accurately report financial relationships with industry inhibits the ability of readers to assess the credibility and risk of bias of publications.

## Abbreviations

AHRQ: Agency for healthcare and research quality; AZ: AstraZeneca; COI: Conflict of interest; CPG: Clinical practice guideline; GSK: GlaxoSmithKline; J&J: Johnson & Johnson; USD: United States dollar.

## Competing interests

SLN has received funding from the US Agency for Healthcare Research and Quality to study conflict of interest in clinical practice guideline development. HKH, LAO, BUB and RF received salary support from the Agency for Healthcare Research and Quality under grant number 1R01HS018500, to study conflict of interest in clinical practice guideline development. All authors declare that they have no other competing interests.

## Authors’ contributions

SLN, HKH and LAO conceived of the study and participated in the design. SLN, HKH, BUB and LAO participated in the collection and management of the data. All authors contributed to the analysis and interpretation of the data and assisted in the preparation, review, and approval of the manuscript.

## Pre-publication history

The pre-publication history for this paper can be accessed here:

http://www.biomedcentral.com/1472-6939/13/24/prepub

## References

[B1] Campbell EG Gruen RL Mountford J Miller LG Cleary PD Blumenthal D A national survey of physician-industry relationships N Engl J Med 2007 356 1742 1750 10.1056/NEJMsa064508 17460228

[B2] Hockenberry JM Weigel P Auerbach A Cram P Hockenberry JM Weigel P Auerbach A Cram P Financial payments by orthopedic device makers to orthopedic surgeons Arch Intern Med 2011 171 1759 1765 10.1001/archinternmed.2011.454 22025434

[B3] Als-Nielsen B Chen W Gluud C Kjaergard LL Association of funding and conclusions in randomized drug trials: a reflection of treatment effect or adverse events? JAMA 2003 290 921 928 10.1001/jama.290.7.921 12928469

[B4] Bekelman JE Li Y Gross CP Scope and impact of financial conflicts of interest in biomedical research: a systematic review JAMA 2003 289 454 465 10.1001/jama.289.4.454 12533125

[B5] Golder S Loke YK Is there evidence for biased reporting of published adverse effects data in pharmaceutical industry-funded studies? Br J Clin Pharmacol 2008 66 767 773 10.1111/j.1365-2125.2008.03272.x 18754841 PMC2675760

[B6] Lexchin J Bero LA Djulbegovic B Clark O Pharmaceutical industry sponsorship and research outcome and quality: systematic review BMJ 2003 326 1167 1170 10.1136/bmj.326.7400.1167 12775614 PMC156458

[B7] Sismondo S Sismondo S How pharmaceutical industry funding affects trial outcomes: causal structures and responses Soc Sci Med 2008 66 1909 1914 10.1016/j.socscimed.2008.01.010 18299169

[B8] Barnes DE Bero LA Why review articles on the health effects of passive smoking reach different conclusions JAMA 1998 279 1566 1570 10.1001/jama.279.19.1566 9605902

[B9] Norris S Holmer H Ogden L Burda B Conflict of interest in clinical practice guideline development: a systematic review PLoS One 2011 6 e25153 10.1371/journal.pone.0025153 22039406 PMC3198464

[B10] Physician Payments Sunshine Act of 2009 http://www.prescriptionproject.org/tools/sunshine_docs/files/0005.pdf

[B11] Dollars for Docswww.propublica.org

[B12] Google Refinehttp://code.google.com/p/google-refine/

[B13] Firebughttp://getfirebug.com

[B14] Adobe Acrobathttp://www.adobe.com/products/acrobat.htm

[B15] Scraping for Journalism A Guide for Collecting Datahttp://www.propublica.org/nerds/item/doc-dollars-guides-collecting-the-data

[B16] Chimonas S Frosch Z Rothman DJ From Disclosure to Transparency: The Use of Company Payment Data Arch Intern Med 2011 171 81 86 10.1001/archinternmed.2010.341 20837820

[B17] Okike K Kocher MS Wei EX Mehlman CT Bhandari M Okike K Kocher MS Wei EX Mehlman CT Bhandari M Accuracy of conflict-of-interest disclosures reported by physicians N Engl J Med 2009 361 1466 1474 10.1056/NEJMsa0807160 19812403

[B18] Bhattacharyya N Lin HW Prevalence and Reliability of Self-Reported Authorship Disclosures in Otolaryngology–Head and Neck Surgery Otolaryngology – Head and Neck Surgery 2009 141 311 315 10.1016/j.otohns.2009.06.010 19716005

[B19] Weinfurt KP Seils DM Tzeng JP Lin L Schulman KA Califf RM Consistency of Financial Interest Disclosures in the Biomedical Literature: The Case of Coronary Stents PLoS One 2008 3 e2128 10.1371/journal.pone.0002128 18461146 PMC2330163

[B20] Uniform requirements for manuscripts submitted to biomedical journalshttp://www.icmje.org/coi_disclosure.pdf

[B21] Krimsky S Rothenberg LS Financial interest and its disclosure in scientific publications JAMA 1998 280 3 225 226 10.1001/jama.280.3.225 9676662

[B22] Chaudhry S Schroter S Smith R Does declaration of competing interests affect readers’ perceptions? A randomised trial BMJ 2002 325 7377 1391 1392 10.1136/bmj.325.7377.1391 12480854 PMC138516

[B23] Schroter S Morris J Chaudhry S Smith R Barratt H Does the type of competing interest statement affect readers’ perceptions of the credibility of research? Randomised trial BMJ 2004 328 7442 742 743 10.1136/bmj.38035.705185.F6 14980983 PMC381324

[B24] Silverman GK Loewenstein GF Anderson BL Ubel PA Zinberg S Schulkin J Failure to discount for conflict of interest when evaluating medical literature: a randomised trial of physicians J Med Ethics 2010 36 5 265 270 10.1136/jme.2009.034496 20448003

